# Modulation of P2X**4/P2**X7/Pannexin-1 sensitivity to extracellular ATP via Ivermectin induces a non-apoptotic and inflammatory form of cancer cell death

**DOI:** 10.1038/srep16222

**Published:** 2015-11-10

**Authors:** Dobrin Draganov, Sailesh Gopalakrishna-Pillai, Yun-Ru Chen, Neta Zuckerman, Sara Moeller, Carrie Wang, David Ann, Peter P. Lee

**Affiliations:** 1 Department of Immuno-Oncology, City of Hope, Duarte, CA, US; 2 Diabetes & Metabolism Research Institute, City of Hope, Duarte, CA, US

## Abstract

Overexpression of P2X7 receptors correlates with tumor growth and metastasis. Yet, release of ATP is associated with immunogenic cancer cell death as well as inflammatory responses caused by necrotic cell death at sites of trauma or ischemia-reperfusion injury. Using an FDA-approved anti-parasitic agent Ivermectin as a prototype agent to allosterically modulate P2X4 receptors, we can switch the balance between the dual pro-survival and cytotoxic functions of purinergic signaling in breast cancer cells. This is mediated through augmented opening of the P2X4/P2X7-gated Pannexin-1 channels that drives a mixed apoptotic and necrotic mode of cell death associated with activation of caspase-1 and is consistent with pyroptosis. We show that cancer cell death is dependent on ATP release and death signals downstream of P2X7 receptors that can be reversed by inhibition of NADPH oxidases-generated ROS, Ca^2+^/Calmodulin-dependent protein kinase II (CaMKII) or mitochondrial permeability transition pore (MPTP). Ivermectin induces autophagy and release of ATP and HMGB1, key mediators of inflammation. Potentiated P2X4/P2X7 signaling can be further linked to the ATP rich tumor microenvironment providing a mechanistic explanation for the tumor selectivity of purinergic receptors modulation and its potential to be used as a platform for integrated cancer immunotherapy.

High extracellular adenosine triphosphate (ATP) is one of the major characteristics of the tumor microenvironment^1^,^2^. Exogenous ATP controls cellular and tissue defense/repair processes via signaling through P1, P2X, and P2Y purinergic receptors and P2X7 signaling has recently been associated with tumor growth and metastasis^3^,^4^,^5^,^6^,^7^. High extracellular ATP levels also occur *in vivo* at sites of trauma, ischemia, or stroke and are associated with massive inflammatory responses and cell death (e.g. in excitable cells such as neurons). Thus, ATP can function as a prototypical danger signal that activates a potent immune response, but can also promote cancer progression. Considering these examples of diametrically opposed functions, ATP/purinergic signaling appears to play a complex role within the tumor microenvironment. Specifically, tumor growth and survival appears to critically depend on optimal extracellular ATP levels that balance tumor-promoting and cytotoxic functions. As such, accumulation of extracellular ATP within the tumor microenvironment is tightly regulated and involves controlled release from the cancer cells as well as degradation by tumor-associated extracellular ATPases such as CD39 and CD73.

ATP associated cell death can involve a signaling pathway downstream of P2X7; its therapeutic potential has been demonstrated in multiple mouse models and clinical trials^4^. However, the use of P2X7 agonists (ATP, ATPγS or Bz-ATP) is limited by systemic toxicity and fails to leverage elevated ATP concentrations found in the tumor microenvironment. In our effort to identify alternative approaches to target this pathway within the tumor microenvironment, we have been studying the commonly used anti-parasitic agent Ivermectin. The anti-tumor activity of both Ivermectin and structurally-related avermectins has been validated in xenogeneic^8^ and immune-competent syngeneic mouse models^9^; in addition, the agents demonstrated broad anti-cancer potential for various solid and hematological malignancies^9^. To explain these activities, several mechanisms have been proposed. These include blockade of MDR exporters and enhanced uptake of doxorubicin/vincristine^10^,^11^, inactivation of PAK1 kinase^12^, and suppression of the wnt/β-catenin pathway^13^. Importantly, avermectins have been shown to exert potent, anti-tumor effects *in vivo* at doses that were subtherapeutic *in vitro*^9^. This implies that the compounds’ actions may be potentiated by the tumor microenvironment and/or the immune system. Congruently, avermectins synergize with chemotherapeutic agents known to induce immunogenic cell death, such as doxorubicin; and Ivermectin stimulates P2X4/P2X7/Pannexin-1 signaling, which augments inflammasome activation in myeloid cells^14^.

Considering these findings, we hypothesized that purinergic signaling may be involved in the mechanism by which Ivermectin kills cancer cells or modulates the immune system. We found that Ivermectin kills mouse and human triple-negative breast cancer (TNBC) cells through augmented P2X7-dependent purinergic signaling associated with caspase-1 and caspase-3 activation. While caspase-3 activity is centrally involved in apoptotic cell death, previous reports indicate that Ivermectin kills leukemic cells in the context of cytosolic Cl^−^ influx and cell size increase; these features suggest another mode of cell death as apoptosis typically involves volume decreases. Since ATP plays a prominent role in regulating cell volume and promotes cancer cell death in response to hypotonic stress^15^, we hypothesized that Ivermectin kills cancer cells in part by enhancing P2X7 sensitivity to ATP. In doing so, the compound appears to induce both an apoptotic and a non-apoptotic, inflammatory type of cell death. The ability of Ivermectin to induce an inflammatory and potentially immunogenic form of cell death is supported by 1) its ability to stimulate autophagy; and 2) recent data showing that ATP release from cancer cells undergoing ICD is mediated by the Ivermectin-sensitive P2X4/P2X7-gated Pannexin-1 channels^16^. Below, we describe Ivermectin’s killing of cancer cells through a mixed apoptotic and necrotic mode of cell death consistent with pyroptosis. Inflammatory and immunogenic forms of cell death are particularly important in the context of cancer types where tumor antigens and therapeutic targets are limited or unknown, such as TNBC, which was used as a model in our studies.

## Results

### Ivermectin kills breast cancer cells through a mixed apoptotic and necrotic mechanism

**Figure 1:**
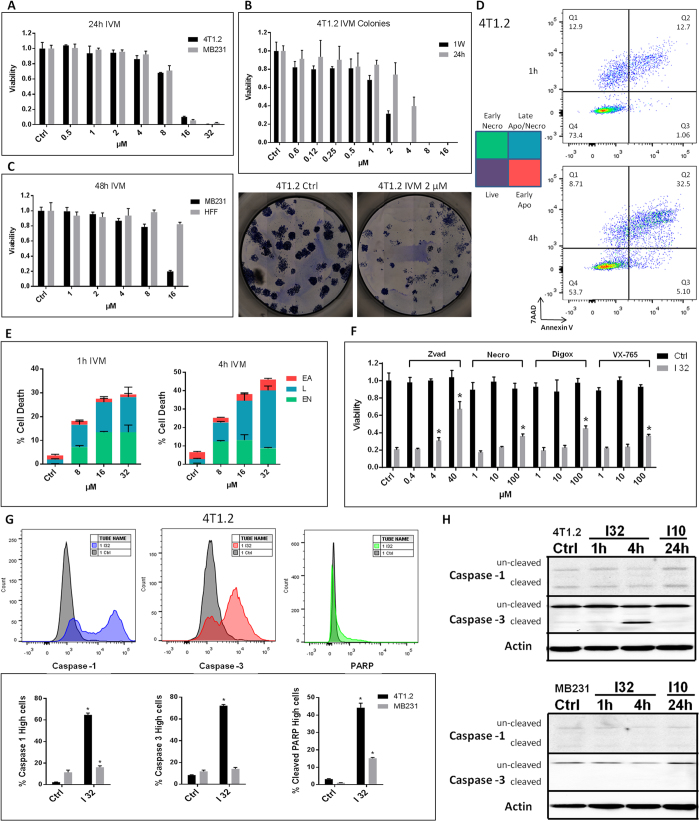
Ivermectin kills breast cancer cells through a mixed apoptotic and necrotic mechanism. (**A**) Mouse (4T1.2) and human (MDA-MB-231) TNBC cells manifest similar sensitivity to Ivermectin. Viability of cells treated with various doses of Ivermectin for 24 h. (**B**) Extended exposure time reduces IC50 values to as low as 2 μM. 4T1.2 cells were seeded at 100 cells/well and individual colonies were counted after a week. Cancer cells were exposed to Ivermectin during the initial 24 h or during the entire duration of the assay. (**C**) MDA-MB-231 breast cancer cells manifest higher sensitivity to Ivermectin compared to normal non-transformed human foreskin fibroblasts (HFFs). (**D**) Flow cytometry analysis showing that cell death proceeds through two distinct pathways: a directly necrotic 7AAD-single positive or Annexin V/PS-single positive apoptotic pathway. (**E**) Kinetics of necrotic versus apoptotic killing of 4T1.2 breast cancer cells. (**F**) Ivermectin-induced cell death can be reversed by inhibition of various controlled cell death pathways. 4T1.2 cells were treated for 4 h with 32 μM Ivermectin in the presence of μM concentrations of Z-vad-fmk, Necrostatin-1, Digoxin, or VX-765, as indicated. (**G**) Activation of Caspase-1, Caspase-3 and cleavage of PARP in 4T1.2 and MDA-MB-231 cells treated with 32 μM for 4h. Asterisk (*) indicates p < 0.05 relative to untreated or Ivermectin alone controls, respectively. (**H**) Western blot analysis showing constitutive and Ivermectin-induced cleavage of caspases 1 and 3 in murine (top) and human (bottom) breast cancer cells.

Due to its ability to stimulate P2X4/P2X7/Pannexin-1 signaling in myeloid cells^14^, we investigated Ivermectin as a prototype agent to modulate purinergic signaling in breast cancer cells. Mouse and human TNBC cells are sensitive to Ivermectin (Fig. 1A), which exhibited IC50 values as low as 2 μM with extended exposure time (Fig. 1B). Consistent with previous findings in leukemia, breast cancer cells manifested higher sensitivity than normal cells to Ivermectin (Fig. 1C). Despite being equivalent at 24 h, the kinetics of Ivermectin killing appears to be faster and more synchronized in mouse compared to human cancer cells. Rapid killing of mouse and human breast cancer cells occurred with Ivermectin doses of 8 μM (and higher), which were used for mechanistic studies *in vitro*. More than 90% of dying cancer cells became directly necrotic (7AAD-positive). The remainder went through an Annnexin V/phosphatidylserine (PS) single-positive apoptotic phase that quickly progressed to secondary necrosis (Fig. 1D,E). Necrosis appears predominant during the first 4 h of treatment, at which point the slower progressing apoptosis gains prominence. The pan-caspase inhibitor Z-vad-fmk inhibited cell death very effectively. The inhibitors of caspase-1 (VX-765), necroptosis (Necrostatin), and autosis (Digoxin) also demonstrated some protection (Fig. 1F). Combinations of inhibitors enhanced protection, which supports the involvement of multiple death pathways (S1A). To clarify, we investigated the downstream mediators of cell death. Cancer cells were treated with Ivermectin and analyzed for 1) PARP cleavage; 2) activation of caspase-1-a characteristic feature of the pyroptotic cell death pathway; and 3) caspase-3 activity typically observed in classical apoptosis. Ivermectin treatment results in rapid cleavage of PARP that occurs in the context of potent caspase-1 and caspase-3 activation (Fig. 1G). While dying cancer cells ultimately manifest cleavage of both caspases, activation of caspase-1 appears to precede that of caspase-3, potentially favoring the necrotic/pyroptotic mechanism of killing (Figure S1B). Interestingly, western blot analysis shows that caspase-1 is constitutively activated/cleaved in breast cancer cells (Fig. 1H), consistent with reports showing constitutive activation of the inflammasome in human melanoma^17^. Our flow data based on fluorescently labeled capspase-1 substrate-specific probe, however, suggest the existence of another level of regulation of caspase-1 activity that can be further enhanced by Ivermectin. Overall, our findings indicate that Ivermectin may kill cancer cells though a mechanism combining apoptosis and necrosis/pyroptosis.

### Role of NADPH oxidases-generated ROS

As described in previous reports, Ivermectin may increase cell volume; affect cytosolic Ca^2+^ flux; and increase production of ROS in leukemic cells^8^. Cell swelling can be mediated by opening of Ivermectin-sensitive Cl^−^ channels, such as the nematode-specific Glutamate-gated chloride channels or mammalian GABA type A and Glycine receptors^18^. According to our results, treatment of mouse 4T1.2 breast cancer cells with Ivermectin resulted in plasma membrane hyper-polarization associated with Cl^−^ influx but modest cell swelling (S2A-C). Cell swelling appeared to be masked by rapid progression to necrotic cell death: the associated loss of membrane integrity and cytosolic content resulted in apparent cell shrinking. Analysis of both PS-positive apoptotic cells and cells surviving the initial killing suggest that death may be preceded by a brief swelling phase. Swelling appears unlikely to be the direct cause of cell death. Osmotic swelling initiates export of Cl^−^ from the cytosol. Specifically, outwardly rectifying I (swell) Cl^−^ channels can be activated by an NADPH oxidases-ROS-H_2_O_2_ mechanism in an ATP release- and P2X4-dependent manner^19^,^20^,^21^. Inhibition of these channels with DCPIB exacerbates Ivermectin killing, indicating that these defensive volume control mechanisms may be indirectly involved in cytotoxicity (S2D).

Since maintenance of cell volume is regulated by ATP-, Ca^2+^- and ROS-dependent mechanisms, we decided to investigate their potential involvement in Ivermectin-induced cell death. Ivermectin induces sustained increases in cytosolic Ca^2+^ that may originate from both extracellular and ER sources and cell death can be suppressed by chelating extracellular Ca^2+^ with EDTA (S2E). In previous studies in leukemic cells, ROS were described as direct mediators of cytotoxicity. In contrast, our findings in breast cancer cells indicate that Ivermectin-induced ROS (S2F) are not the direct cause of cell death. The ROS scavengers, NAC and GSH, effectively blocked H_2_O_2_-generated ROS (Figure S2G) but were less effective against Ivermectin-induced ROS and completely failed to prevent, or even delay, Ivermectin-induced killing (Figure S2H).

**Figure 2:**
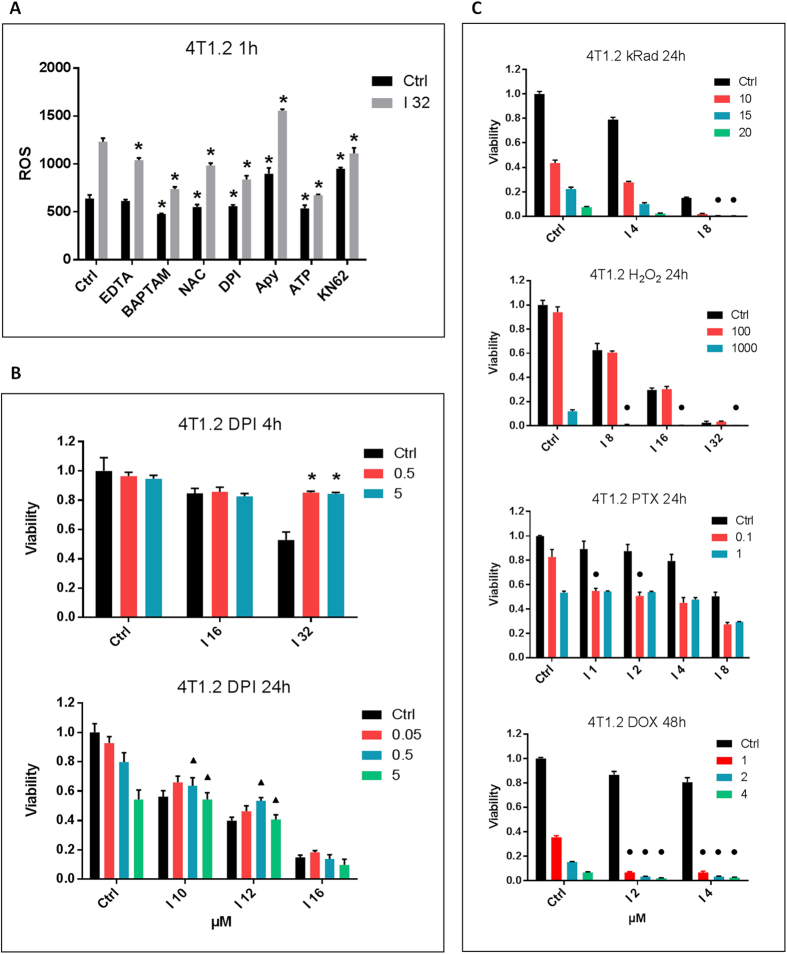
Role of NADPH oxidases-generated ROS. (**A**) Ivermectin-induced ROS are Ca^2+^–, ATP-, and P2X7-regulated. 4T1.2 cells were labeled with a ROS detection probe and treated for 1 h with 32 μM Ivermectin in the presence of 1 mM EDTA, 5 mM NAC, 5 μM DPI, 2500 μunits/ml Apyrase, 3 mM ATP, and 10 μM KN-62. (**B**) Ivermectin-induced cell death is transiently reversed by inhibition of NADPH oxidases with DPI (μM concentrations as indicated). Triangles (▲) indicate significant antagonism CI > 1.0. (**C**) Synergy between Ivermectin and H_2_O_2_- or irradiation-generated ROS, as well as the ROS-inducing chemotherapeutic agents paclitaxel (PTX) and doxorubicin (DOX). 4T1.2 cancer cells were irradiated (10–20 kRad) or treated with H_2_O_2_ (10–1000 μM), PTX (0.1–1 μM), or DOX (1–4 μM) and incubated with Ivermectin for 24h/48h. Circles (●) indicate significant synergy CI < 1.0, see Table 1. Asterisk (*) indicates p < 0.05 relative to untreated or Ivermectin alone controls, respectively.

Overall, our findings suggest that it is not the magnitude of Ivermectin-induced ROS that kills cancer cells. Unlike ROS of mitochondrial origin, NADPH oxidases-generated ROS are known to play important signaling roles and some NADPH oxidases are also Ca^2+^-dependent. We demonstrated that Ivermectin-induced ROS are regulated partially by Ca^2+^, NADPH oxidases, ATP and P2X7 (Fig. 2A). Of note, an inhibitor of NADPH oxidases, Diphenyleneiodonium (DPI), is able to transiently block Ivermectin-induced killing (Fig. 2B). Short-term exposure to DPI and high doses of Ivermectin were found to be more informative since NADPH oxidases are essential for tumor cell growth and prolonged exposure to DPI exacerbates oxidative stress and becomes directly toxic. These data suggest that Ca^2+^/NADPH/ROS signaling is directly involved in the mechanism of killing. Importantly, Ivermectin was found to be synergistic with irradiation, H_2_O_2_-generated ROS, and chemotherapeutic agents that are known inducers of ROS stress such as doxorubicin and paclitaxel (Fig. 2C, Table 1). This is consistent with previous data in leukemia showing synergy between Ivermectin and the ROS-inducing chemotherapeutic drugs doxorubicin and cytarabine^8^. Although NADPH oxidases-generated ROS can contribute to ROS imbalance and ROS-mediated killing, the magnitude of Ivermectin-induced ROS does not appear to be directly cytotoxic. Therefore, we hypothesized that cell death is driven by ROS-dependent signaling pathways.

### Dual roles of ATP and purinergic signaling in Ivermectin’s killing

**Figure 3:**
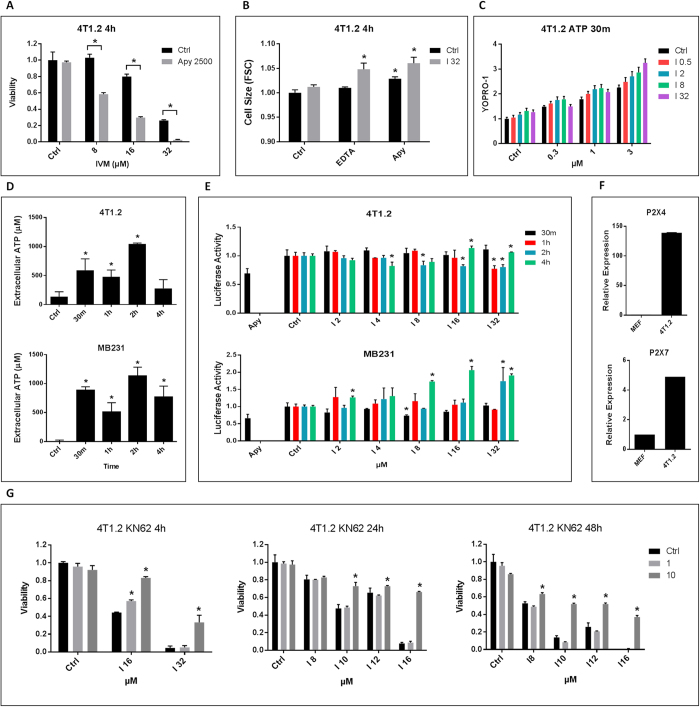
Dual roles of ATP and purinergic signaling in Ivermectin’s killing. (**A**) Depletion of extracellular ATP with Apyrase (2500 μunits/ml) exacerbates killing of 4T1.2 cell by Ivermectin. (**B**) Extracellular Ca^2+^ and ATP contain Ivermectin-induced cell swelling. 4T1.2 cells were treated with 32 μM Ivermectin for 4 h in the presence of 1 mM EDTA or 2500 μunits/ml Apyrase. (**C**) Ivermectin (0.5–32 μM) and high concentrations of extracellular ATP (0.3–3 mM) synergistically open pannexin-1 channels and permeabilize the membrane on live cells (7AAD-positive dead cells were gated out). 4T1.2 cells were treated for 30 min as indicated in the presence of 7AAD and 5 μM YOPRO-1. (**D**) Analysis of supernatants from Ivermectin-treated murine and human TNBC cells showing rapid release of ATP followed by its transient depletion. Depletion of extracellular ATP with Apyrase (2500 μunits/ml) was used as a positive control. (**E**) Analysis of membrane-proximal ATP levels using cancer cells engineered to express a membrane-bound Luciferase. (**F**) qPCR demonstrating over-expression of P2X4 and P2X7 receptors in mouse 4T1.2 breast cancer cells versus mouse embryonic fibroblasts (MEF). (**G**) The P2X7-specific inhibitor KN-62 (1–10 μM) blocks Ivermectin cytotoxicity (IVM 8–32 μM, 4–48 h treatments). Asterisk (*) indicates p<0.05 relative to untreated or Ivermectin alone controls, respectively.

Ivermectin has been shown to enhance P2X4-dependent purinergic signaling in myeloid cells, driving a ROS-dependent pathway that normally activates the cytosolic NLRP3 inflammasome^14^,^22^,^23^,^24^,^25^,^26^. Since volume control depends on ATP release and Ca^2+^ signaling, we investigated the role of ATP and purinergic signaling in Ivermectin-induced killing. Ivermectin killing was initially enhanced by both depleting extracellular ATP with Apyrase (Fig. 3A) and blocking purinergic signaling with a non-specific inhibitor of P2 receptors (Suramin) (Figure S3A). Ivermectin-induced cytotoxicity was suppressed in multiple cancer cell lines by blocking extracellular ATPases (CD39 and CD73) with PSB 069 (Figure S3B). This confirms an initial protective role of extracellular ATP and implicates ATP and ATP-dependent Ca^2+^/ROS signaling in the early defense mechanisms that contain Ivermectin-induced killing. We also showed that extracellular Ca^2+^ and ATP prevent Ivermectin-induced cell swelling, which provides a mechanistic explanation for their initial protective role (Fig. 3B).

The protective effect of the ATP/Ca^2+^/ROS-pathway is, however, only transient: extended exposure to either high concentrations of ATP or sustained elevation of cytosolic Ca^2+^ is cytotoxic to both mouse and human cancer cells. We decided to investigate whether the sustained increase in cytosolic Ca^2+^ levels described earlier was preceded by extracellular ATP release from non-necrotic viable cells. ATP release from the P2X4/P2X7-gated Pannexin-1 channels is a potent feed-forward mechanism operating in autophagic cancer cells and activated leukocytes. Ivermectin synergized with ATP in opening P2X4/P2X7/Pannexin-1 channels resulting in partial permeabilization of the plasma membrane of 4T1.2 cells to YOPRO-1 without compromising cell viability (Fig. 3C), consistent with previous reports on Ivermectin activity in myeloid cells^14^. Blocking Pannexin-1 channels with either a Panx-1 mimetic peptide or Probenecid enhanced Ivermectin cytotoxicity validating the initial protective role of ATP release (Figure S3C). Analysis of supernatants from TNBC cells demonstrated an immediate increase in extracellular ATP levels in response to Ivermectin, followed by a period of its transient depletion (Fig. 3D). Using cells engineered to express a membrane-bound form of Luciferase, similar results were obtained by measuring plasma membrane-proximal extracellular ATP levels (Fig. 3E). These findings suggest that ATP release is necessary to balance an increased demand/consumption of extracellular ATP. Following the transient decline during the protective phase, ATP levels are restored and continue to rise. This is consistent with a possible switch to a cytotoxic role for ATP during Ivermectin-mediated killing (Figure S3D,E).

A significant body of literature indicates that cancer cells are sensitive to high concentrations of extracellular ATP^1^,^3^. Ivermectin can enhance signaling of the P2X4 receptor, which can complex with both pannexin-1 and death-mediating P2X7 receptors^27^,^28^. Therefore, we hypothesized that Ivermectin may modulate the sensitivity of cancer cells to ATP. We demonstrated up-regulation of P2X7 and Ivermectin-sensitive P2X4 receptors in 4T1.2 cells (Fig. 3F) as well as in other murine cancer cell lines (Figure S3F). We also validated over-expression of the P2X4 and P2X7 receptors as a characteristic feature of human breast cancer lines (Figure S3G). Importantly, the sensitivity of different human cancer cell lines to Ivermectin was found to correlate with their sensitivity to ATP (Figure S3H). We found that the semi-specific P2X7 inhibitor KN-62 was effective at blocking Ivermectin-induced necrotic and apoptotic cell death (Figs 3G, S3I). Most importantly, Ivermectin induced a P2X7-dependent death pathway in a broad spectrum of murine and human cancer cell types (Figure S3J). Surprisingly, we did not observe any protection using other P2X7 receptor-specific inhibitors such as PPADs, OxATP, and A438079 (Figure S3K). The ATP-gated P2X7 receptors possess three independent activities: 1) opening of intrinsic Ca^2+^ channels; 2) modulation of Pannexin-1 channel activity and 3) activation of a cell death pathway. These appear to be mediated by different parts of the molecule and may be modulated by different inhibitors. To test whether the P2X7-specific inhibitors are in fact affecting P2X7-dependent mechanisms (rather than working through off-target effects), we evaluated their ability to block membrane permeabilization. Both KN-62 and A4308079 suppressed permeabilization to YOPRO-1 in a dose-dependent fashion (Figure S3L). Moreover, the doses of KN-62 required to effectively block the pannexin-1 channel were consistent with doses required to protect against Ivermectin-induced killing. Paradoxically, A438079 effectively blocked permeabilization but could not prevent Ivermectin-induced cell death, suggesting that membrane permeabilization and cell death might be two distinct P2X7-dependent pathways. This is also supported by the fact that Pannexin-1-dependent ATP release itself can be transiently protective. Thus the ultimate role of the P2X4-P2X7-Pannexin-1 channel activity may depend on the magnitude and duration of channel opening.

### Excessive CaMKII signaling and MPTP contribute to cell death

**Figure 4:**
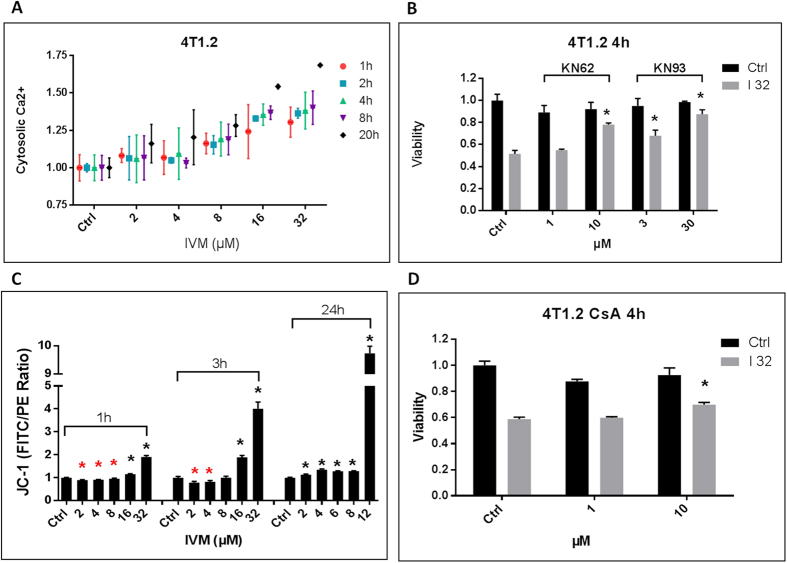
Excessive Ca^2+^/CaMKII signaling and MPTP contribute to cell death. (**A**) Kinetics of cytosolic Ca^2+^ increase in 4T1.2 cells treated with Ivermectin. (**B**) Inhibition of CaMKII is equally effective at blocking the initial cytotoxicity of Ivermectin. 4T1.2 cells were treated for 4 h with 32 μM Ivermectin in the presence of the P2X7/CaMKII dual inhibitor KN-62 or the CaMKII-specific inhibitor KN-63, at μM concentrations as indicated. (**C**) Flow cytometry analysis of JC-1 loaded 4T1.2 cells showing that high doses of Ivermectin result in a sudden mitochondrial de-polarization, while lower doses cause similar effects but after transient hyper-polarization. (**D**) MPTP contributes to Ivermectin. 4T1.2 cells were treated for 4 h with 32 μM Ivermectin in the presence of the MPTP inhibitor Cyclosporin A at μM concentrations as indicated. Asterisk (*) indicates p < 0.05 relative to untreated or Ivermectin alone controls, respectively.

KN-62 is not completely specific for the P2X7 receptor and also weakly inhibits Ca^2+^/Calmodulin-dependent protein kinase II (CaMKII). More than 1 μM of KN-62 is needed to protect against Ivermectin-induced cell death; and this value is consistent with KN-62’s inhibition of CAMKII. CaMKII activity correlates with sustained increase in cytosolic Ca^2+^ (Fig. 4A) and is known to be a downstream target of P2X7 receptor signaling and to mediate neuronal cytotoxicity in the context of I/R, stroke and neurodegenerative diseases, pathologic conditions where ATP-mediated excitotoxicity is known to play a prominent role^29^,^30^. We investigated whether Ivermectin-induced cell death was mediated by P2X7, CaMKII, or a combination of both. To this end, we used a CaMKII-specific inhibitor (KN-93) while knocking down the P2X7 receptor with shRNA. KN-93 effectively blocked Ivermectin-induced cell death, confirming that over-activation of the CaMKII underlies the initial wave of cell death in many Ivermectin-sensitive cancer cell lines (Figs 4B, S4A). Reports indicate that the combination of Ca^2+^ and ROS-mediated oxidation results in Ca^2+^-independent constitutive activation of the CaMKII kinase that initiates the highly inflammatory cell death pathway in I/R injury and stroke^31^,^32^,^33^. This is consistent with our own data showing protection through inhibition of NADPH oxidases. We also demonstrated that while lower doses of Ivermectin cause mitochondrial hyperpolarization, massive cancer cell death occurs in the context of a sudden depolarization of mitochondria (Fig. 4C) and that blockade of mitochondrial permeability transition pore (MPTP), the downstream effector of this cell death pathway, is able to confer partial protection against Ivermectin (Figs 4D, S4B).

### CaMKII-independent P2X7-mediated killing

**Figure 5:**
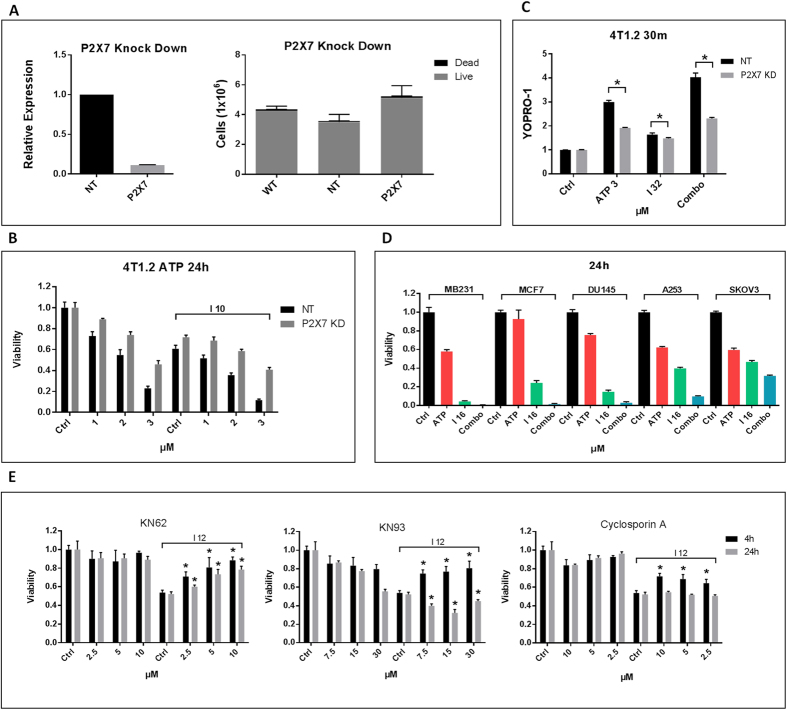
CaMKII-independent P2X7-mediated killing. (**A**) qPCR showing the extent of gene knockdown in 4T1.2 cells transfected with shRNA targeting the P2X7 receptor versus non-targeting (NT) control (left panel). The right panel shows that P2X7 knockdown does not compromise cancer cell growth and viability. One million wt, shRNA(NT) and shRNA(P2X7) 4T1.2 cells were plated and incubated for 48h before evaluation of cell numbers and viability. (**B**) P2X7 knockdown cells are more resistant to ATP and the ATP/IVM combo. 4T1.2 cells were treated for 24 h with 1–3 mM ATP, 6–10 μM Ivermectin, or combinations of ATP and Ivermectin. P2X7 knockdown suppresses the synergy between Ivermectin and high dose ATP, as shown by calculation of combination index (CI) values, see Table 2. (C) P2X7 knockdown cells are resistant to ATP/Ivermectin induced membrane permeabilization (Asterisk (*) indicates p < 0.05). (**D**) The combination of Ivermectin (16 μM) and exogenous ATP (3 mM) can synergistically kill even some resistant human cancer cell lines like breast (MCF7), prostate (DU-145), head and neck (A253), and ovarian (SKOV3) cancer cells. (**E**) Comparison of the protective effects of P2X7, CaMKII, and NADPH oxidases inhibition in short-term (4 h, 32 μM IVM) and long-term (24 h, 12 μM IVM) exposure of 4T1.2 cells to Ivermectin. Asterisk (*) indicates p < 0.05 relative to Ivermectin alone controls.

Given the low affinity of P2X7 for ATP, high extracellular ATP concentrations are needed for P2X7 receptors to deliver death signals. As extracellular ATP is transiently depleted and protective during Ivermectin-induced cell death, we sought to clarify the way in which the P2X7 pathway is activated and involved in cytotoxicity. To this end, we compared the ability of Ivermectin to kill wild type and P2X7-knockdown 4T1.2 cells (Fig. 5A). Overall, P2X7 knockdown cells were more resistant to ATP and ATP/Ivermectin cytotoxicity (Fig. 5B, Table 2), which is consistent with a pivotal role for the P2X7 receptor in ATP-mediated cytotoxicty. In addition, the P2X7 knockdowns were more resistant to ATP- and ATP/Ivermectin-induced membrane permeabilization to YOPRO-1 (Fig. 5C), suggesting that over-activation of this protective mechanism might lead to cytotoxicity. High concentrations of extracellular ATP kill cancer cells in a P2X7-dependent fashion but with much slower kinetics compared to Ivermectin. Therefore, in addition to targeting ATP/P2X4/P2X7 signaling, the drug likely affects other pathways. We show that Ivermectin and ATP can synergistically kill even ATP- and Ivermectin-resistant human cancer cell lines (Fig. 5D). Interestingly, the P2X7 receptors appear to be involved in both the early necrotic and later apoptotic mechanisms of killing (Figure S5A). We further compared the ability of CaMKII/MPTP and P2X7 inhibition to provide short-term (4 h) versus long-term (24 h) protection against Ivermectin. While inhibition of NADPH oxidases, CaMKII, and MPTP provided significant protection against Ivermectin cytotoxicity within the first 4h, only KN-62 was able to provide long-term protection against Ivermectin and ATP that extended up to 24 h (Figs 5E, S5B,C). At this time point, NADPH oxidases, CaMKII and MPTP inhibitors were not only ineffective but could even enhance cell death. Thus the P2X7/CaMKII inhibitor KN-62 rather uniquely confers long-term protection by blocking both the initial CaMKII-mediated cell death as well as the later P2X7-mediated killing. The early necrotic pathway induced by Ivermectin appears to progress within the first few hours of treatment and is effectively blocked by inhibitors of NADPH oxidases, CaMKII, and MPTP, consistent with massive Ca^2+^ overload. This fast death pathway is likely initiated by early ATP release and P2X7-dependent caspase-1 activation, but can be transiently delayed by depletion of extracellular ATP, which allows for the slower progressing caspase-3-dependent apoptotic pathway to become prominent. The transiently protective functions of extracellular ATP during this phase can be mediated by the P2X4/P2X7/Pannexin-1-dependent ATP release or by other P2X/P2Y receptors, particularly those involved in osmotic cell volume control. Of note, while ATP is transiently protective, the combination of ATP and Ivermectin seems to favor the necrotic over apoptotic mechanism of killing (Figure S5D), further implicating ATP/P2X7 signaling in both the early necrotic and delayed apoptotic cell death. Consistent with the hypothesis that the P2X7 and CaMKII death pathways partially overlap is our finding that CaMKII appears to be critically important for ATP and ATP/Ivermectin-induced membrane permeabilization (Figure S5E). Thus the balance between the apoptotic and necrotic death pathways downstream of the P2X7 receptor appears to be regulated by the complex interplay between extracellular ATP, ROS levels, and CaMKII activation.

### Ivermectin induces autophagy

**Figure 6:**
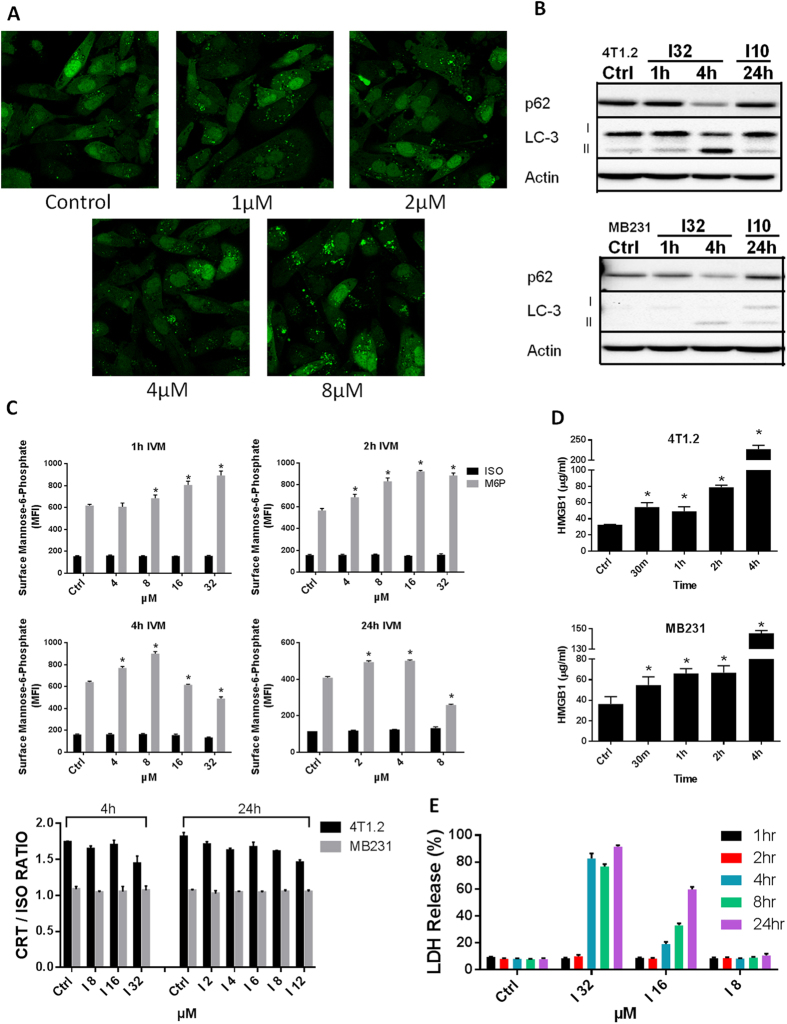
Ivermectin induces autophagy. (**A**) Ivermectin induces autophagy in breast cancer cells. MDA-MB-231 GFL-LC3 cells were treated with different doses of Ivermectin for 24 h and formation of green fluorescent puncta was evaluated by confocal fluorescence microscopy. (**B**) Western blot confirming the induction of autophagy in both murine and human breast cancer cells, as evidenced by LC3 lipidation and autophagic degradation of p62(SQSTM1). (**C**) Ivermectin up-regulates surface exposure of M6P receptor (top panel) but does not impact the exposure of Calreticulin (CRT) (bottom panel). MDA-MB-231 cells (triplicates) were treated with different doses of Ivermectin for up to 24 h and surface stained with antibody-specific for the human M6P receptor versus isotype control. Similarly, 4T1.2 and MB231 cells were treated with different doses of Ivermectin for 4–24 h, and were stained with an antibody specific for both human and mouse CRT versus isotype control. Mean fluorescence intensity (MFI) values and ratios versus isotype control were calculated after gating on live/membrane-intact cells. (**D**) Ivermectin (32 μM) induces release of HMGB1 from murine and human TNBC cells (triplicates). (**E**) Ivermectin treatment induces release of cytosolic LDH, data are normalized to maximum release (lysis). Asterisk (*) indicates p < 0.05 relative to untreated or Ivermectin alone controls, respectively.

The mixed apoptotic and necrotic mechanism of killing prompted us to analyze Ivermectin’s ability to modulate some features associated with immunogenic cell death: HMGB1/ATP release and the surface exposure of Calreticulin (CRT). Release of ATP and surface exposure of CRT on apoptotic cells have already been linked to autophagy and ER stress^16^,^34^,^35^. We demonstrate that Ivermectin is a potent inducer of autophagy (Fig. 6A,B), consistent with its ability to stimulate ATP release and P2X4/P2X7/Pannexin-1 membrane permeabilization. Moreover, while Ivermectin does not significantly impact surface CRT (S6A,B, Fig. 6C, bottom panel), its ability to up-regulate plasma membrane exposure of mannose-6-phosphate (M6P) receptors could potentially render cancer cells susceptible to bystander CTL/NK cell-mediated killing, as described recently (Fig. 6C, top panel)^36^,^37^. Ivermectin also appears to be a potent inducer of HMGB1 release (Fig. 6D), preceding that of LDH (Fig. 6E), consistent with both necrosis and reports describing the key role of caspase-1 in regulating non-classical secretion of HMGB1^38^,^39^.

## Discussion

Accumulating evidence suggests that long-term clinical responses in some patients after chemotherapy involve host anti-cancer immune responses; one mechanism is the induction of immunogenic cell death (ICD) by several chemotherapeutic agents including doxorubicin and oxaliplatin^33^. ICD is characterized by surface exposure of calreticulin and the release of ATP and HMGB1. These hallmarks of ICD have been linked to ROS-related ER stress and the induction of autophagy^40^,^41^. ATP and HMGB1 mediate immunostimulatory functions in cardiac infarction and brain stroke, where ischemia/reperfusion (I/R) injury is associated with massive inflammatory responses and necrotic cell death through P2X7-dependent purinergic signaling and NLRP3/caspase-1-dependent pyroptosis. Pyroptosis is an important defense mechanism that might have evolved to protect myeloid and epithelial cells against certain intracellular parasites including viruses and bacteria (e.g. Salmonella and Listeria)^42^,^43^. Components of activated inflammasomes can be secreted and re-captured by other myeloid cells, providing a potent amplification step stimulating protective immunity^44^,^45^. Caspase-1 has also been reported to regulate non-classical secretion of HMGB1 and serves as a link between HMGB1 and purinergic signaling^39^. ATP and HMGB1 function as prototypic danger signals that can promote immune mediated destruction as well as inflammatory-reparative responses^46^,^47^. The interplay between ATP and HMGB1 appears to be essential for tumor growth, angiogenesis and metastasis.

Hypoxia and the maintenance of elevated levels of extracellular ATP and HMGB1 are characteristics shared between the tumor microenvironment and sites of I/R injury. However, it is still unclear how tumors manage to effectively utilize the tumor promoting functions of the ATP/HMGB1/caspase-1 system while successfully evading immune-mediated destruction. Cancer cells of diverse origin are known to be very sensitive to high concentrations of extracellular ATP. This is likely due to the expression of higher levels of P2X7 receptors that have been correlated with tumor survival, progression and metastasis^7^,^48^,^49^ and can be potentially linked to the constitutive activation of the inflammasome pathway in cancer^17^. Apart from being directly cytotoxic, high extracellular ATP concentrations can break local immunosuppression and promote inflammation, which is prevented by extracellular ATPases (CD39/CD73) degrading ATP to immunosuppressive adenosine. Tumor growth and survival is therefore critically dependent on an optimal balance between the pro-survival and cytotoxic functions of purinergic signaling and caspase-1. A recent report showed that pharmacological stimulation of purinergic signaling can, indeed, induce pyroptosis of cancer cells^50^. The sensitivity of tumors to ATP and agonistic analogues has been extensively investigated but the therapeutic window for such systemic approaches might be limited by toxicity and lack of tumor specificity^1^. To circumvent this issue, we proposed to modulate purinergic signaling with agents that enhance the sensitivity of the P2X4/P2X7/Pannexin-1 complex to ATP. Such an approach capitalizes on the high levels of extracellular ATP characteristic of the tumor microenvironment, thus minimizing availability and systemic toxicity issues.

**Figure 7:**
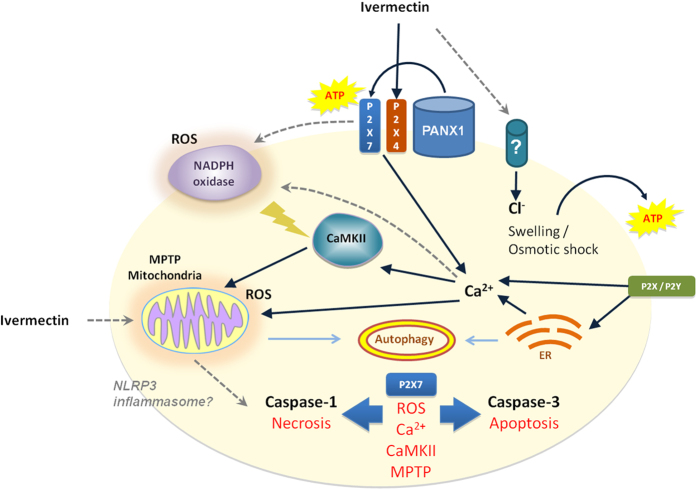
Model of P2X4/P2X7/Pannexin-1-induced cancer cell death. Ivermectin induces P2X4/P2X7-dependent activation of Pannexin-1 channels and release of ATP. The release of ATP might be transiently protective, but only in cell types that are highly sensitive to Ivermectin-induces cell swelling when ATP and Ca^2+^ signaling are essential for control of cell volume. In cancer cells where no cell size changes can be observed (for example human TNBC MDA-MB-231 cells), high concentrations of ATP (1–3 mM) immediately enhance Ivermectin cytotoxicity. Potentiated P2X7 receptor signaling drives a fast progressing necrotic/pyroptotic mechanism driven by NADPH oxidases-generated ROS, cytosolic Ca^2+^/CaMKII activation, and MPTP, and characterized by caspase-1 cleavage, due to possible NLRP3 inflammasome activation. Necrotic killing is followed by a slower progressing apoptotic cell death program mediated by caspase-3 activation. The failure of the default apoptotic pathway might be attributed to faster activation of caspase-1, inadequate autophagic control of mitochondrial MPTP, collapse of cellular energy metabolism, resulting in rapid progression of necrotic cell death. Damage to mitochondria and ER stress as well as potential depletion of cellular ATP reserves simultaneously promote autophagy that might render even the slower apoptotic pathway immunogenic.

Ivermectin has recently been shown to have anti-tumor properties that we hereby link to its ability to augment P2X4/P2X7/Pannexin-1 signaling and caspase-1 activation, which is also associated with cancer cells’ elevated expression of P2X4/P2X7 receptors. Paradoxically, Ivermectin appears to effectively eliminate tumors *in vivo* at much lower doses that are non-toxic to cancer cells *in vitro*^9^. Our finding that Ivermectin potentiates P2X4/P2X7 purinergic signaling in cancer cells correlates to the ATP rich tumor microenvironment, thus providing a mechanistic explanation for this phenomenon. The enhanced tumor specificity of Ivermectin can also be attributed to the Warburg effect and the high dependence on glycolysis under the hypoxic tumor environment *in vivo*. Caspase-1 has multiple downstream effector mechanisms^51^,^52^ that include cleavage of key enzymes in the glycolytic pathway^53^. Such activity could potentially compromise cellular energetics, ER Ca^2+^ ATPases, and cytosolic Ca^2+^ homeostasis^54^, resulting in failure of the default immunologically silent apoptotic pathway and the prevalence of CaMKII/MPTP-mediated necrotic cell death^55^. In support of this hypothesis, we show that Ivermectin-induced cell death is consistently mediated through the P2X7, CaMKII and MPTP pathways in all mouse and human cancer cell lines tested (Fig. 7). Ivermectin directly impacts the balance between ATP-dependent pro-survival and cytotoxic signals, converting a key pro-survival and tumor-promoting pathway into a P2X7- and caspase-1-mediated immunogenic cell death. P2 purinergic receptors provide essential anti-apoptotic and pro-survival Ca^2+^-mediated signals to cancer cells allowing them to overcome various therapeutic or environmental stresses. ATP-dependent Ca^2+^ signaling also appears to be involved in regulation of cell volume. Exposure of cancer cells to hypotonic stress causes cell swelling and release of ATP that can subsequently kill through a P2X7-dependent mechanism^15^, indicating that overstimulation of otherwise protective mechanisms can ultimately result in cytotoxicity. P2X7 receptors and CaMKII mediate trophic and protective purinergic/Ca^2+^ signaling in various cell types, but the same signaling cascade can promote both survival and cell death dependent on signaling strength or duration. Thus, CaMKII/MPTP-mediated necrotic cell death appears to result from over-stimulation of the P2X7 pathway. Limited MPTP could be contained by Ca^2+^-induced autophagic removal of damaged mitochondria and stressed ER; whereas a massive collapse of mitochondria and mtDNA-stimulated NLRP3 inflammasome activation would lead to necrosis. Such a massive mitochondrial collapse followed by autophagic vacuolization was indeed observed in Ivermectin-treated MCF7 breast cancer cells and has been reported in the literature for MCF7 cells in which autophagy was induced by exposure to ATP^56^. Interestingly, the inhibitor of autosis digoxin conferred partial protection against Ivermectin and a recent report described a novel P2X7-mediated autophagic cell death of dystrophic muscle cells^57^, prompting further validation of the precise mode of Ivermectin-induced necrotic cell death. Definitive validation of the pyroptotic nature of cell death is particularly problematic in view of recent findings showing that caspases-11(4/5), rather than NLRP3/caspase-1, might be indispensible for execution of pyroptosis^58^, consistent with our own data showing incomplete protection with Caspase-1-specific inhibitors VX-765 and ZYVAD-fmk.

Our mechanistic studies suggest that the anti-cancer properties of Ivermectin can be potentiated by therapies inducing autophagy, ATP release, and ROS, such as irradiation and doxorubicin. Ivermectin-induced ROS were initially believed to directly mediate killing of leukemic cells. Recently, ATP-regulated ROS were shown to play roles in inflammation, NF*k*B signaling, and recruitment of leukocytes^59^. Thus, purinergic-regulated ROS might be involved in maintenance of tissue homeostasis in response to cellular stress or to directly promote cancer cell growth and survival. Further investigation is necessary to identify the precise sources of Ivermectin-induced ROS with particular focus on the role of purinergic receptors and Ca^2+^-responsive NOX family members Nox5, Duox1, and Duox2^60^. NADPH oxidases-generated ROS regulate cytoskeleton organization and multiple cytosolic kinases/phosphatases, including CaMKII, which could provide further insight into the mechanism by which Ivermectin down-regulates the PAK1 and beta-catenin pathways. Beta-catenin, for example, appears to be a direct target of inhibitory phosphorylation by CaMKII^61^.

In summary, Ivermectin promotes a novel form of cancer cell death involving a combination of enhanced autophagic apoptosis and highly inflammatory regulated necrosis, consistent with pyroptosis. The induction of autophagy is particularly interesting since it represents an important cellular defense mechanism against various chemotherapeutics, while enhancing immunogenicity and rendering cancer cells susceptible to immune mediated killing^36^,^37^. The P2X4/P2X7/Pannexin-1 system regulates ATP release from autophagic cancer cells but also functions as a positive feedback/amplification step that is involved in the recruitment and activation of T cells, macrophages and dendritic cells^62^,^63^. The anti-cancer properties of Ivermectin are therefore not limited to direct cytotoxicity and require further investigation of its immunomodulatory potential and impact on cancer immunotherapy. Calreticulin (CRT) exposure data present in Figure 6 were generated using two CRT-specific monoclonal antibodies (Abcam ab22683 and ab83220), both of which did not demonstrate a significant effect of Ivermectin on CRT exposure. We recently repeated these assays using a polyclonal rabbit anti-CRT antibody (Abcam, ab2907), which is more commonly used in the literature. This antibody demonstrated that Ivermectin was indeed able to up-regulate exposure of CRT on the surface of live murine (4T1.2, CT26) as well as human (MDA-MB-231) breast cancer cells prior to apoptosis (Supplementary Figure 7).

## Materials and Methods

### Cell lines

4T1.2 Balb/c triple-negative breast cancer cells were obtained from Robin Anderson, PhD, Peter MacCallum Cancer Centre, Melbourne, Australia. The human breast cancer cell lines MDA-MB-231(triple-negative), MCF7 (ER-positive), SKBR3 (Her-2-positive) were obtained from Robert Hickey, City of Hope National Medical Center. The murine B16 (C57BL/6 mouse melanoma), CT26 (Balb/c colon adenocarcinoma), and DDHer2 (Balb/c Her-2/neu breast carcinoma) cell lines were obtained from Glenn Dranoff, MD, Dana-Farber Cancer Institute, Boston. Human melanoma (A2058, A375) and pancreatic cancer (PANC1, MiaPaca-2) lines were a gift from Don Diamond, City of Hope National Medical Center. Human Prostate (DU145), Head and Neck (A253) and Leukemia (MV411) cells were obtain from Marcin Kortylewski Lab, City of Hope National Medical Center. All cancer cell lines were grown in RPMI medium supplemented with 10%FBS, Pen/Strep, Glutamine, and HEPES Buffer (R10).

### Reagents

The following reagents were used: Ivermectin (Sigma I8898-250MG), KN-93 (Tocris, 5215-1 MG), Cyclosporin A (Tocris, 1101–100 MG), KN-62 (Tocris, 1277-1 MG), PSB 069 (Santa Cruz, sc-204216 10 MG), DCPIB (Tocris, 1540–10 MG), A438079 (Tocris, 2972–10 MG), z-vad-fmk (Enzo Life Sciences, ALX-260-020-M001-1 MG), vx765 (InvivoGen, inh-vx765-1-10MG), oxATP (Sigma Aldrich, A6779–25MG), PPAD (Sigma Aldrich, P178–10MG), DPI (Sigma Aldrich, D2926–10 MG), NAC (Sigma Aldrich, A9165–25 MG), Suramin (Sigma Aldrich, S2671–100 MG), Necrostatin (Enzo Life Sciences, BML-AP309–0020–20 MG), Digoxin (Sigma Aldrich, D6003–100 MG), Apyrase (New England Biolabs, M0393S–10,000 milliunits), ATP (Sigma Aldrich, A2383–1G), Pannexin-1-Inhibitor (AnaSpec, 61911–1 MG), Probenecid (Sigma Aldrich, P8761–25 G), YOPRO-1 Iodide (Life Technologies, Y3603-1 mL), GSH (Sigma Aldrich, G4251–1 G).

### Cell viability

Cancer cells were plated on 96 well flat-bottom plates and allowed to attach overnight. Cells were then pre-incubated with drugs for 1 h and treated with Ivermectin for 4 h. In the case of 24 h and 48 h incubations the cells, Ivermectin and drugs were plated together. All treatments and controls were done in triplicate wells and results were validated in at least two independent experiments. Following treatment the plates were washed once with PBS and cell viability was measured using the acid phosphatase assay. Briefly, cells were incubated for 1h at 37 °C in 100 μL assay buffer (0.16 M sodium acetate, 0.1% Triton-X-100, supplemented with 4-Nitrophenyl phosphate disodium salt hexahydrate, Sigma Aldrich, at 0.00263 g/ml). Reaction was stopped using 10 μL of 1N sodium hydroxide. Absorption at 405 nm was measured within 10 min using a multi-well plate reader. Mode of cell death and viability of cells in suspension was evaluated using flow cytometry. Briefly, cancer cells were pre-incubated with drugs for 30 min to 1h, as indicated, treated with Ivermectin for 4 h in 5 ml FACS tubes, washed twice in PBS and stained with PE-conjugated Annexin V and 7AAD (1 μl per 100 μl/200,000 cancer cells) for 15 min.

### Cytosolic Cl^−^ influx

Cancer cells in suspension were treated with various doses of Ivermectin in R10 medium for 1h at 37 °C and 5% CO_2_. Cells were then washed with PBS and loaded with 10 μM SPQ probe (Life Technologies, M440) in a hypotonic buffer containing 50% PBS and 50% double distilled water. Following brief 15 min incubation at room temperature in the dark cells were washed once with PBS and immediately analyzed by flow cytometry measuring fluorescence in the AF350 channel.

### Cytosolic Ca^2+^ flux

Cancer cells in suspension were washed with PBS and loaded with 1 μM Rhod-3 AM (Life Technologies, R10145) for 30 min at room temperature in the dark. Cells were washed with PBS couple of times and treated with various doses of Ivermectin in R10 medium. Cells were incubated for 15 min to 4 h in 5 ml FACS tubes at 37 °C and 5% CO_2_, followed by immediate flow cytometry analysis of fluorescence in the PE channel. Chelating of extracellular and intracellular Ca^2+^ was performed by supplementing the Ivermectin treatment medium with 1 mM EDTA (USB Corp, 15694) or by adding 20 μM BAPTA-AM (Tocris, 2787) to the Rhod-3 AM loading solution, respectively.

### Mitochondrial membrane potential

Cancer cells in suspension were washed with PBS and loaded with 2 μM JC-1 (Life Technologies, T3168) for 15 min at room temperature in the dark. Cells were washed twice with PBS and treated with various doses of Ivermectin in R10 medium. Cells were incubated for 1 h/4 h in 5 ml FACS tubes at 37 °C and 5% CO_2_. After 1 h/4 h of treatment cells were analyzed by flow cytometry. Mitochondrial polarization was evaluated by measuring the corresponding changes in fluorescence in the FITC and PE channels. Treatment with 50 μM CCCP (Sigma C2759–100 MG) was used as a positive control.

### Plasma membrane polarization

Cancer cells in suspension were treated with various doses of Ivermectin in the presence of 0.5 μM DiBAC_4_ (3) (Sigma D8189). Treatments were performed in serum free RPMI medium and cells were analyzed by flow cytometry after 1 h of incubation. De-/hyper-polarization results in increased/decreased fluorescence in the GFP channel, respectively.

### Reactive oxygen and nitrogen species

Cancer cells in suspension were washed with PBS and loaded with 50 μM H2DCFDA (Molecular Probes, Life Technologies, D399) or 5 μM DAF-2 (Cayman Chemical, Catalog # 85165) for 30 min at room temperature in the dark. Cells were washed twice with PBS and treated with various doses of Ivermectin in R10 medium. Cells were incubated for 1 h in 5 ml FACS tubes at 37 °C and 5% CO_2_, and immediately analyzed by flow cytometry. Generation of intracellular ROS and NO results in corresponding increases in the GFP fluorescence of the H2DCFDA and DAF-2 probes, respectively.

### Caspase activation and PARP cleavage

Cancer cells in suspension were treated with Ivermectin in 5 ml FACS tubes for 4 h. Analysis of caspase activation was done using the FLICA® 660 Caspase 3/7 Assay Kit (ImmunoChemistry Technologies 9125) and the FAM FLICA™ Caspase 1 Assay Kit (ImmunoChemistry Technologies 98), following manufacturers’ instructions. PARP cleavage was analyzed after fixing the cells (eBioscience, Cat# 00–5123 and Cat# 00–5223) for 30 min at RT. Cells were then washed twice with permeabilization Buffer (eBioscience, Cat# 00-8333) and then stained with FITC-conjugated antibody specific for cleaved PARP (10 μl per 100 μl, BD Biosciences 558576) for 1hr at RT. Cells were washed twice with permeabilization buffer, followed by flow cytometry analysis.

### Generation of P2X7 receptor knockdown 4T1.2 cells

4T1.2 cells were transduced using Sigma mission shRNA lentiviral transduction particle with four P2X7 shRNA (NM_011027/TRCN0000068568, NM_011027/TRCN0000068570, NM_011027/TRCN0000068571, NM_011027/TRCN0000068572) or Non-Target control (SHC002V). The viral particles were added to growing cells in the presence of 8 μg/ul polybrene (Sigma, St Louis, MO), and incubated overnight at 37 °C. Puromycin selection (2000 ng/ml) was done from third day onwards to select for stably transformed cells.

### Extracellular ATP release

4T1.2 and MDA-MB231 cells were plated onto 96 well flat-bottom plates at a density of 10,000 cells per well and incubated overnight before transfection with pMetLuc-Mem luciferase vector, following manufacturers’ instructions (Clontech Laboratories Inc). Forty eight hours post transfection, stable transformants were selected using G418 (1000 μg/ml). The cancer cells engineered to stably express the membrane-bound Luciferase were treated with Ivermectin for 30 min-4 h and luminescence was measured in the presence of 2 μM Luciferin. ATP concentration in Ivermectin treated supernatants was measured using ATP determination kit (Life Technologies, A22066) following manufacturers’ instructions.

### Real-time RT-PCR analysis

Total RNA was isolated from cancer and normal cell using Qiagen micro RNA isolation kit according to manufacturer’s instructions. The cDNA were synthesized from 1μg of total RNA using SuperScript II reverse transcriptase kit, (Invitrogen Inc) and oligo dT. Using mouse gene specific primers for P2X7 (forward 5′-TGGATGACAAGAACACGGATG-3′ and reverse 5′-CAGGATGTCAAAACGGATGC-3′) and 18s RNA primers as controls, real-time RT-PCR was performed in duplicate for each sample in an iCycler (BioRad). Reactions (25 μl) contained cDNA template (1 μl), primers and SYBR green PCR mix (Applied Biosystems). Relative quantification was done by the ΔΔCT method (–).

### Surface exposure of CRT and Mannose-6 Phosphate receptor

Surface exposure of Calreticulin (CRT) and Mannose-6 Phosphate receptor (CD220) was evaluated by staining with monoclonal antibodies specific for CRT (unconjugated, specific for both mouse and human, Abcam ab22683, and PE-conjugated, specific for human only, Abcam, ab83220) and CD220 (Thermo Schientific, MA1-10148) versus isotype controls. Staining for CRT was performed as previously described^64^. Cell viability/membrane integrity probe was used to discriminate/gate between live and dead cells (Life Technologies, L23105).

### HMGB1 and LDH Release

HMGB1 and LDH release were evaluated using an HMGB1 detection kit (IBL International, ST51011) and the CytoTox 96 Non Radioactive Cytotoxicity Assay (Promega, G1780), following manufacturers’ instructions.

### Western Blotting

Protein lysates from tumor cells treated with Ivermectin were prepared using RIPA buffer. Activation and cleavage of caspases was evaluated using antibodies specific for caspase-1 (NOVUS, NBP1–45433) and caspase-3 (Cell Signaling, 9665). Induction of autophagy was validated using antibodies specific for p62/SQSTM1 (American Res., 03-GP62-C) and LC3 (Cell Signaling, 4599).

### Statistical Analysis

Statistical analysis of the data was performed using two-tailed unpaired Student’s t-test. Figures depict data representative of at least two independent and similar experiments. Statistically significant differences (P < 0.05) are shown with asterisks (*).

## Additional Information

**How to cite this article**: Draganov, D. *et al.* Modulation of P2X4/P2X7/Pannexin-1 sensitivity to extracellular ATP via Ivermectin induces a non-apoptotic and inflammatory form of cancer cell death. *Sci. Rep.* **5**, 16222; doi: 10.1038/srep16222 (2015).

## Supplementary Material

Supplementary Information

## Figures and Tables

**Table 1:** Synergy between Ivermectin and ROS.

CompuSyn CI values	<0.8 Synergistic		0.8–1.2 Additive	>1.2 Antagonistic	
	**I 4**	**I 8**		**I 8**	**I 16**
kRad 15	1.16	0.75	**H**_**2**_**O**_**2**_ **100**	1.46	1.39
kRad 20	0.94	0.63	**H**_**2**_**O**_**2**_ **1000**	0.24	0.20
	**I 1**	**I 2**		**I 2**	**I 4**
PTX 0.1	0.35	0.52	**DOX 1**	0.43	0.61
PTX 1	1.30	1.51	**DOX 2**	0.42	0.56

**Table 2:** Effect of P2X7 knockdown on the synergy between Ivermectin and ATP.

CI values	<0.8 Synerg	0.8-1.2 Addit	>1.2 Antag
4T1.2 shRNA (P2×7)			
	**I 6**	**I 8**	**I 10**
ATP 1	1.49	1.39	1.35
ATP 2	1.18	1.03	1.11
ATP 3	0.96	0.91	0.86
4T1.2 shRNA (NT)			
ATP 1	1.18	1.01	1.24
ATP 2	1.17	1.01	1.10
ATP 3	0.67	0.64	0.63
