# Supplementary material for: Modulation of P2X4/P2X7/Pannexin-1 sensitivity to extracellular ATP via Ivermectin induces a non-apoptotic and inflammatory form of cancer cell death
Source: Sci Rep. 2015 Nov 10;5:16222. doi: 10.1038/srep16222 (PMC4639773; doi:10.1038/srep16222)
Supplement: Supplementary Information [file srep16222-s1.doc]

**Modulation of P2X4/P2X7/Pannexin-1 sensitivity to extracellular ATP via Ivermectin induces a non-apoptotic and inflammatory form of cancer cell death**

1Dobrin Draganov, 1Sailesh Gopalakrishna-Pillai, 2Yun-Ru Chen, 1Neta Zuckerman, 1Sara Moeller,

1Carrie Wang, 2David Ann, and 1Peter P. Lee*

1Department of Cancer Immunotherapeutics & Tumor Immunology (CITI), City of Hope, Duarte, CA;

2 Diabetes & Metabolism Research Institute, City of Hope, Duarte, CA.

**Supplemental data**

Figure 1.


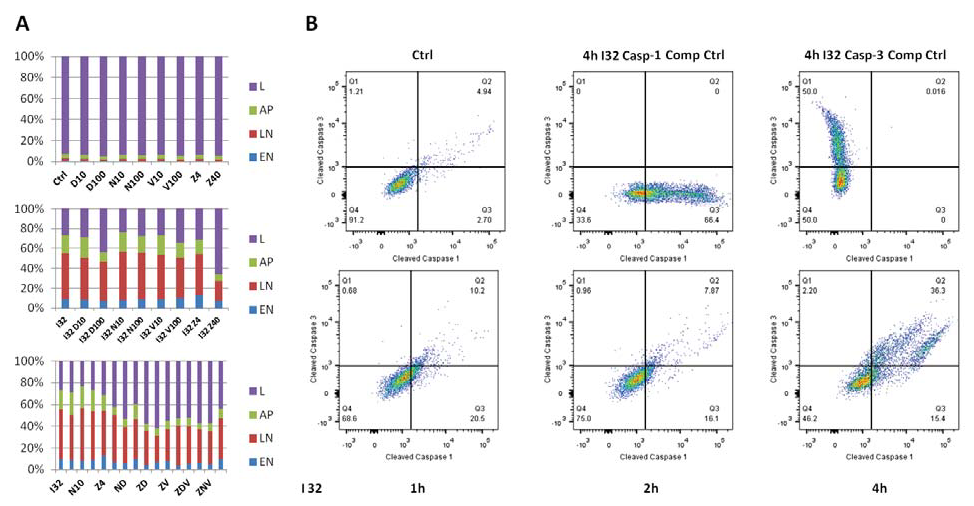


Figure 2.

A **B** D


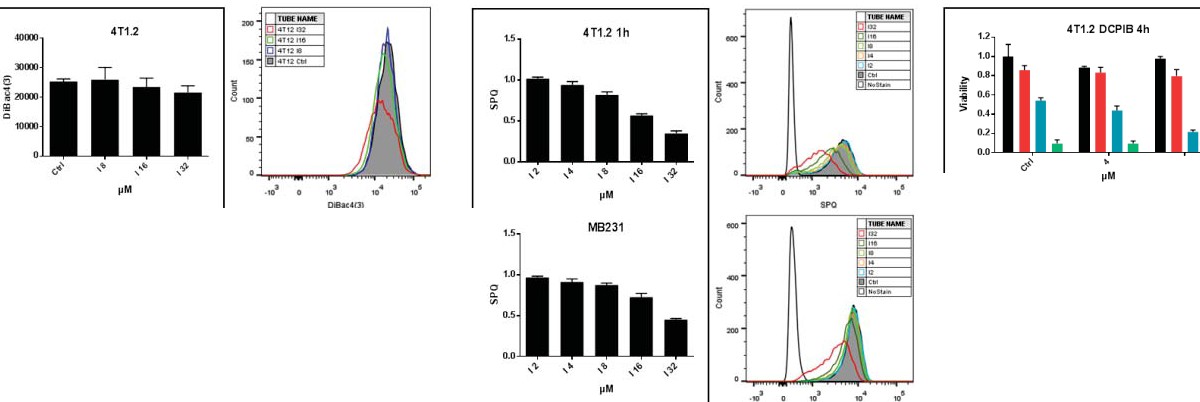


-_ "'

-_ ,1 ,6

i :d M0231

.... ! -

**{; MB2J10CPIB4h**

0.>.

•u

0

*O't:-* ... ,,•,.....

,.

c

•' . •' .· •'

***<7'*** ,... ,.


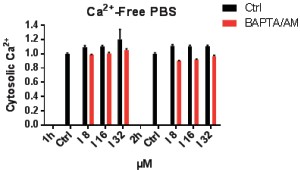
E

Singlets:All

E.


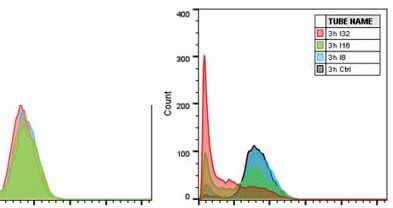


!-

E.


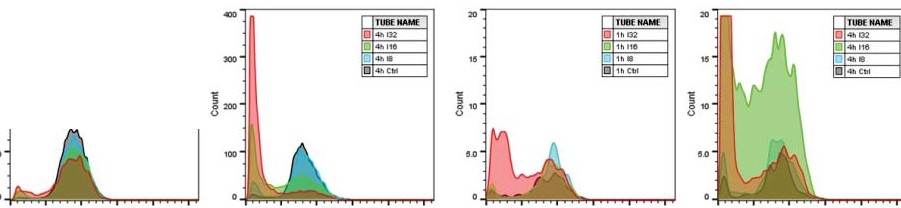


Singlets:Annexin V+/Apoptotic

m..


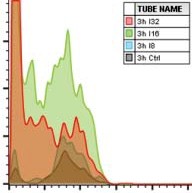


!· !·

,... ,...

o''-Ri< h R10ModiumlllUc

*c*:..

....

§A/AM

*"'<f ,.t; (},.t; ..(}_-'}-*

!-

-­

'"""


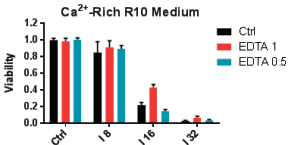
....

E.


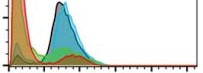

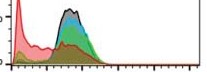

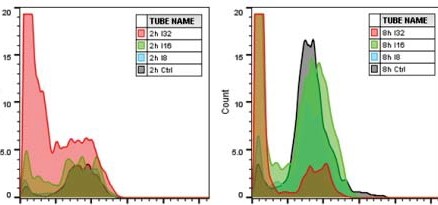


!- !-

F G **H**


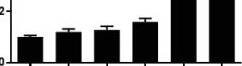

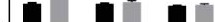

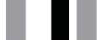

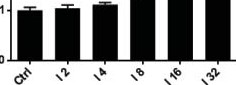

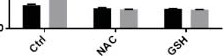

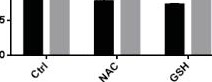

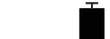


4T1.2 ROS **4T1.2 4T1.2**

B. '


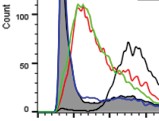
'"""

- -

"lli**4**J**T1.2 4h** ill

- Hf>l -1 32

- "" - "'"

" i1..

0

,.

'

*l' ...*

1..

s ..

*I' ,<t* . *#* •••

•Orl

**• 1n**

, ,•

....

,,. ,,

*1'4> #*

*+- '*

*,<t*

MB231ROS **MB231 MB231**

"U**4T1.22**i**4h** ll

• em •c •Orl

-132 **t • 116**

"0

....

- Hj)2

...

s ..

i ..

• *#"./'#*

*#*·'"'

Figure 3.

A B

1.2

4T1.2 Suramin


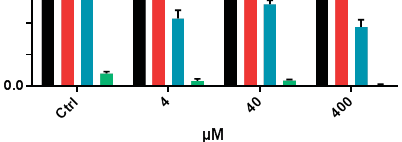


-arl

1.2


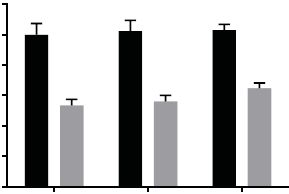


MB231 PSB 4h

1.2

4T1.4 PSB 4h

•ctrl

1.0

,?0.8

0.6

- 1 8

- 116

- 1 32

0.8

0.6

1.0

1.0 -1 32

0.8


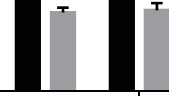


:g 0.6

5 o.4

0.2

> 0.4

0.2

:;: 0.4

0.2

c

4T1.2 Panx1


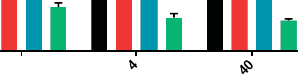


12

1n

- Ctrl 0.8

•I S

0.0

1.2

1.0

*".$-* .... ,

M

RMI PSB4h

0.0

1.2


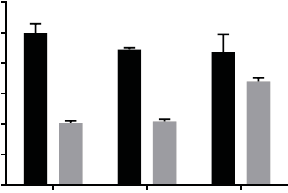


1.0

0.8

*d''* .... ....

M

DDHer2 PSB 4h

- 11 6 :g 0.6

*OB*

:g 0.6

:g 0

> *OA*

*02*

- 1 32 > 0.4

0.2

0.0

r-, n

M

> 0.4

0.2

0.0

.... ....

*on*

12

1n

*OB*

.il'

0

"'

M

4T1.2 Probenecid

D

.0


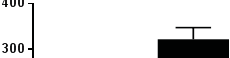

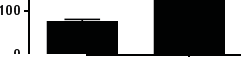


.*e*^0^.

'-"' ....

30m

'-"'

M

30m

> *OA*

*02*

0


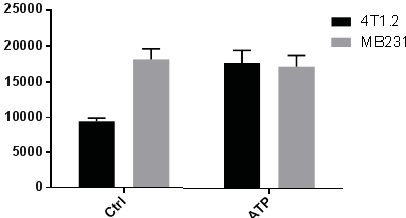
ii 0

..

0:

*on*


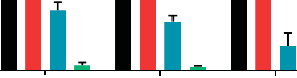


"' ,<S> *<S>*

'

M

E

0

0

>

•"'}

F


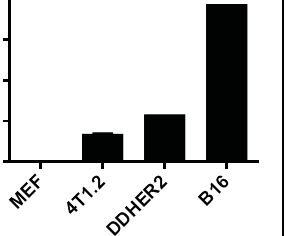


>

,,

"'"'

G

| GSE414458C0atillstt T.TTEST Ave;Norm Avt6C P2)(Rs P2R)(1 U9E·45 7.90 6.95 | |
| --- | --- |
| , | P21UO 0.1710 6.07 5.66  ..118!-1 706 74  P2R)(6 0.0969 6.79 6.48  P2RX7 UlE-6 447 5.09  P2RY1 0.160 4.27 3.71  P2RV2 7.01f.5 6.86 7.62  P2RY4 0.466 5.75 5.87  P2RY12 0.319 3.66 3.71  LTIAFt 0.0211 6.55 6.79  lf'AR6 0.841  G PIU7 0.887 5.04  P2RYS 0.01132 5.13 5.48  LPAR4 0.408 31>5 )_ 77  OXGJtl 6.94(-3 4.50 5.00  NOX4 2.93E-3 471 3.89  NOX3 0.397 4.70 4.72  NOX2 0 120 5.35 4.92  DUOXl 1.01£-3 6.21 5.45  DUOX2 4.71E-3 564 5.06  GABRAl 0.738 4.56 4.34  0.152 5.39 5.22  GAIIRA4 *0.041* B6 5.10  4.52  GLRA1 0.0841 5.82 4.51  0.166 7.29 |
| P2YRs |
| NOXs  G A BA Rs |
| Gly<ine Rs |

1.2

0.8 -ct

4T1.2 ATP 4h

c 1000

P2X4

1.0

-

:E 0.6 1 32

> 0.4

*·u;*

Q"a)'.

X

LU

"i

800

600

400

0.2

0.0

li n fi n

1i 200

0::

0

v... ' .,

M

1.2

1.0

0.8

:E 0.6

> 0.4

0.2

0.0

MB231ATP 4h

v..v. .,


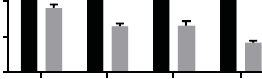


M

c 30

*·u;*

"'

Q)

20

Q.

X

LU

10

"i

1i

0::

0

P2X7

,...,...'); "'""'


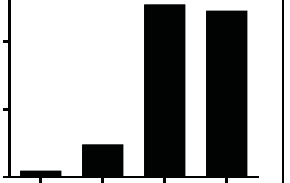


1> *i-;*

()()

Figure 3 (cont.).

**H J**

1.2

1.0

:?' 0.8

1..i 0.6

> 0.4

0.2

ATP IVM 24h Sensitivity

Ctrl KN-62


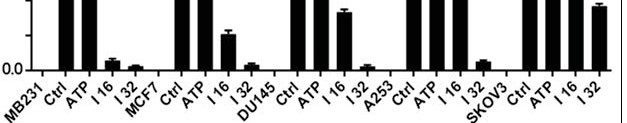


**Humn Cll Lines**


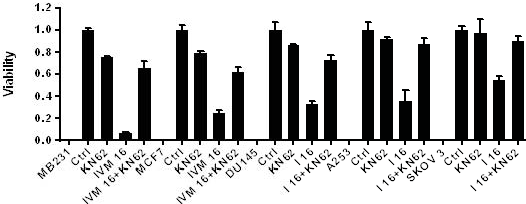


**Humn Cell Lin es**

,0.2.


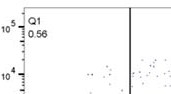

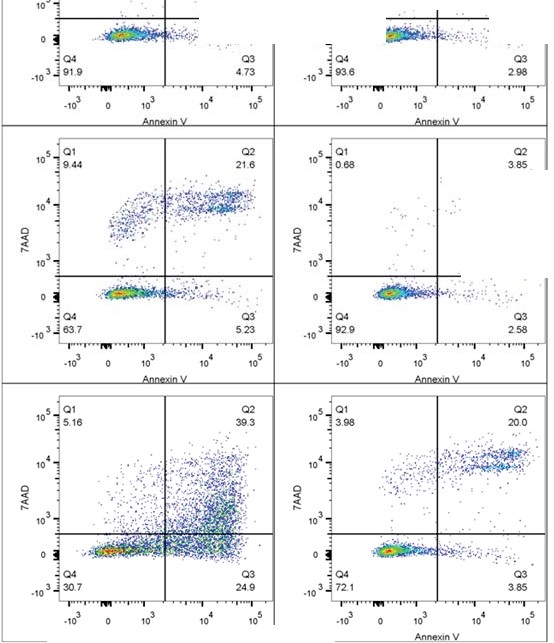


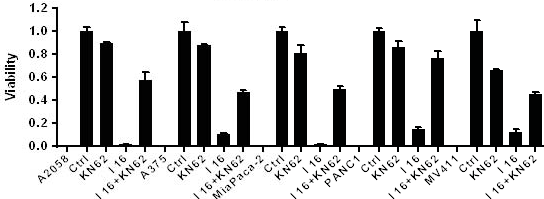
• ra;- 02

10 **los1** 200

Ctrl

··;

.1

" l

'.

...l

...

.•

.·..


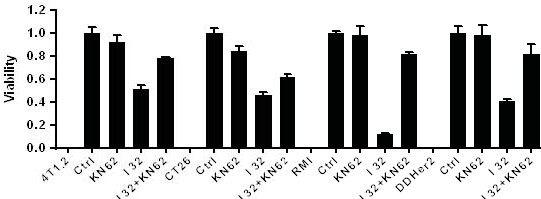
**Murin e Cell lin es**

18

132

_-v

: ,.:..: .. '..

... \

**K**

4T1.2 A438079

12 - eM

**!**'**lll.. llt n. u...** =: l;

**v** " ...... ***"t::::J***

f **lllltlj;'·' Ii; IIt**

**L**

40000 132+ATP


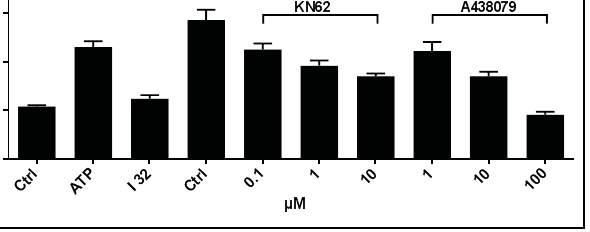
30000

20000

10000

0.0

'il

I I 1 I

**v ' ..... "t:::J *"t:::>t::::J***

**1**

**11.1. 1i.;·i;_ n.**

**:**

Figure 4.


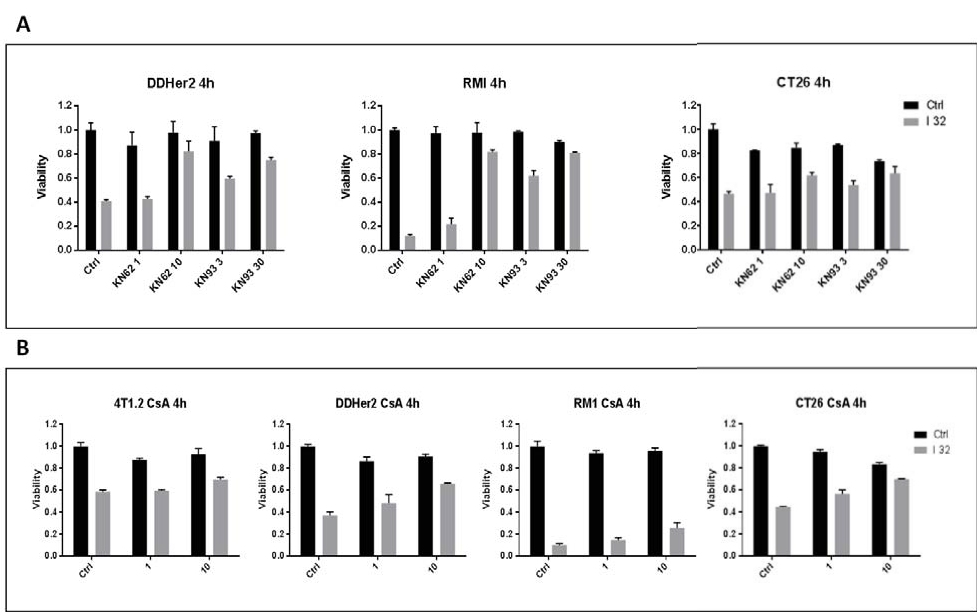


Figure 5.

A

132 lM

,• 01

..760

4T1.2 shRNA(P2X7}


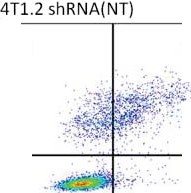


511 ...

02 ,• 01 02


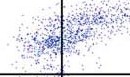


"'

,•

E

4T1.2 30 min Ctn

-

-

KN62

lh

•'

o•

·•' 773

·•'

,•

03 o• 03


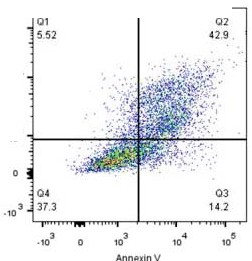
•' .· •' ·•' •' .· •'


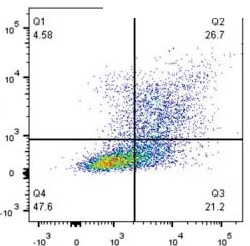


"' · U1 122

,•

"

2.5

2.0

61.5

a:

•·0.·511 1

KN93

Suramin

i

<.q

0.0

,•

2h

v-s.'

.",.'q

'""' ")<'('t-

'

B


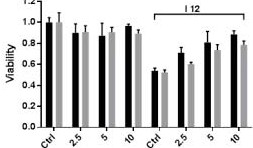
4T1.2 KNU

c


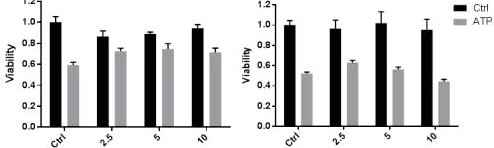
MB231K-N6·2h _,.,


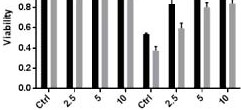


4T1.2 KNU

MB231 KN6Z


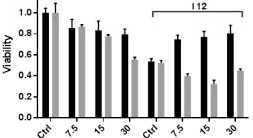

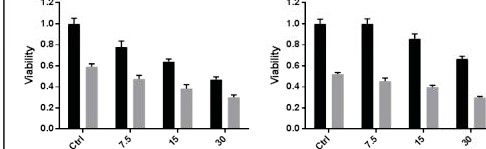
4T12. K N13

MB231 KNU


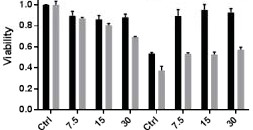
'"

4T1.2KNU MB231 KNt:J


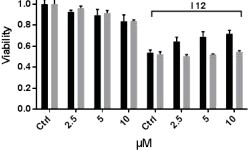
4T1.2 Cycol sporin A

MB231 Cyclospor nA


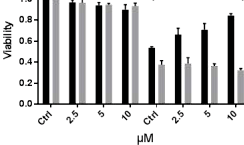
"'

4T1.2Cyclospor nA MB231cyclospor n A

'J O,.l·: 10.$ :

5 o•

> o.•

0.2 0 2

..0.1.. 02

0.0 0.0

*<l "ll .. ... <. l'* ...

'M

01 Ol ..01 "


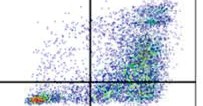


Ctrl ..

·

...."' ..

'' •."' "'

..'" '"

•' .-....

•' "'

.·

·•' •' •'

•' ..

•' "'

.·

·•' •'


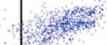


•' •'

•' •' 103


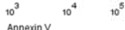


.· ..

·•' •'

-··

ATP .·'" '·"

.

.·'"

-· -·

"' •·"' '"

•'


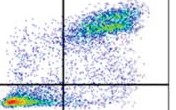
.·"'

:.4 -(T-'i

,·1·.·*_.:..* .

•' . - ..

*,(-*

•' •'

*.:.*

•' '" ...

.

" .. " .. ..

.. •' '

' •'

•' "'

"' •'

'" .· "'

•' "' "'

•' •-' · •'

• .•

•' •-' · • -·

**Figure 6.**

**A**

m-[]- OJ- DJ

4T1.2 CT26 MB231

. . -

!. !. **J•**

Control : : •

[-J-w- -w-

... •' ***J*** ... . . ... . .... ***J*** •• .. ... ... •' .. •' ... •'

.

-

!" !•

*(*

6tM /24h : : :

. -

-.w..... **,.t** -_, ... ,.•

·..w.... **,.t** -... -... .[.. [-***J*** ..· ......... ..

!. !. !.

**B** :::::::t. . :....._ r... ... .r.. •r......J_ ...... •' .**0 ...** m... *.._...* ... •'

Live:[[]Dead:[jJ Live:[]Dead.:GJ

|  | **l"** | **l.** |  | ! | !.. |
| --- | --- | --- | --- | --- | --- |
| **4T1.2** | • | .. | **MB231** | • | .. |

Control :1... ... ... ... ... ...

.... .. ... ... ... ...

Control :

..

...

•' ... •' •'

,.....· ... •' ..· ...

: M/4hI... ...i.. ..J. ... ..

I.....i... •J' ... •'

: M/4h{.. •'I*,.,* •J' .. •' {.. •' .. ]•' ... ...

""M/4h ""M/4h

{1] {[]

... •' ***J*** •' ..• •' ... •' •' •' ..• •'

{.....•I... •J' ..• •'

I.....•i•' •J' ..• ...

- - - -

uv..UJo..d.liJ Liv• [[D••d.:w

**l"** !" **l**.

!.".

**4T1.2** • " **MB231** • ..

!:[[-

..

:

Control ,.....· ... ... ... ...

, .......· ...

Control , ...·

.. ...

, ...... •' ..· ...

l:[J-

... •'

IVM

.. ./ •'

!::1.1

...•. ... •' ..• •'

:•w• · ..-......· •'

:.w.. ***.I*** -***.....•••***

-..w.. •' -...... •'

12 *""I*24:f... •'D......... •'

-

. \ . ..

• 12 J.IM *I* 24h • •

- -

... •' .. ....... •' .....· ***J*** ... ... ... •...· ...-•' ..· •'


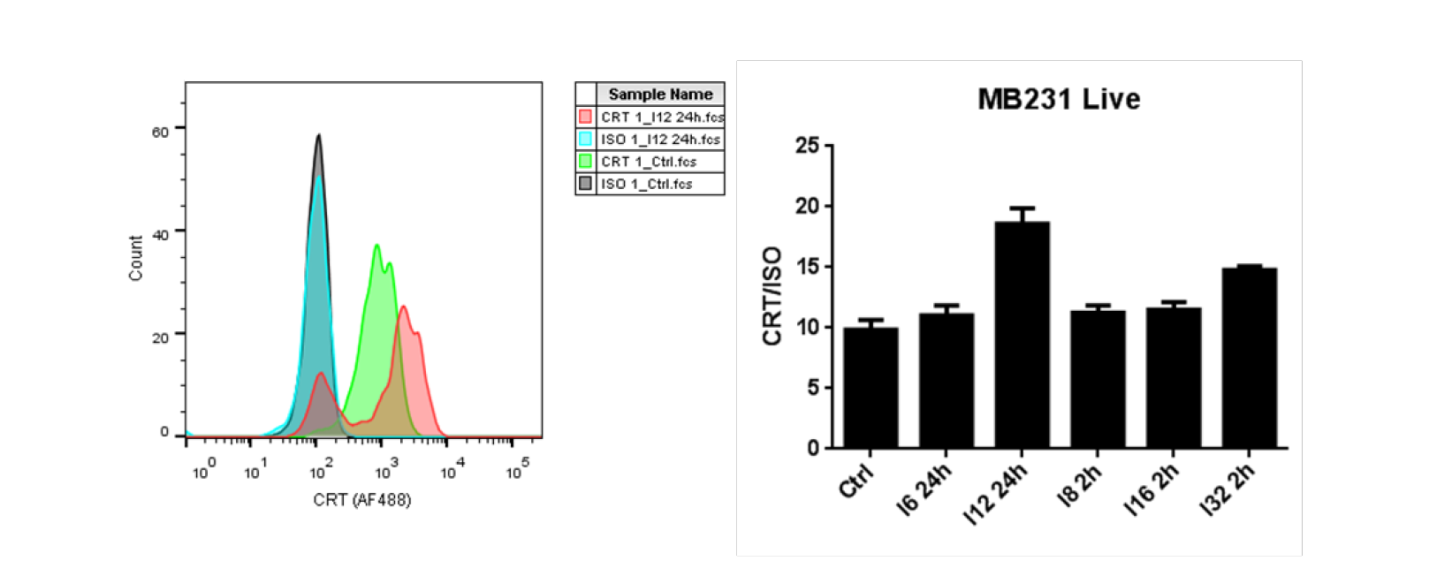
**Figure** **7**

**Supplemental Figures**

**Figure 1. Ivermectin kills breast cancer cells through a mixed apoptotic and necrotic mechanism.** (A) Combinations of different cell death inhibitors provide superior protection against Ivermectin. 4T1.2 cells were treated with 32 PM Ivermectin for 4h and flow cytometry was used to evaluate the fraction of live, apoptotic, early and late necrotic cells. Cells were also pre-incubated for 1h with different inhibitors of cell death pathways: apoptosis (z-vad-fmk, 4 and 40 PM), necroptosis (necrostatin-1, 10 and 100 PM), autosis (digoxin, 10 and 100 PM), and pyroptosis (VX-765, 10 and 100 PM). Synergy between the inhibitors was evaluated using the less effective lower drug dose. (B) Flow cytometry analysis of

caspase-1/3 activation in 4T1.2 cells treated with 32 PM Ivermectin for 1h-4h showing that caspase-1 cleavage precedes that of caspase-3.

**Figure 2. Role of NADPH oxidase-generated ROS.** (A) Ivermectin causes hyper/de-polarization of the

plasma membrane of mouse and human breast cancer cells, respectively. (B) Ivermectin promotes slightly bigger cytosolic Cl- influx in mouse compared to human breast cancer cells. (C) Ivermectin causes very modest swelling of apoptotic and surviving 4T1.2 cells. (D) Ivermectin-induced cell death is exacerbated by inhibition of I (Cl,swell) channels with DCPIB. (E) Ivermectin induced Ca^2+^ flux in 4T1.2 cells is dependent on both extracellular and intracellular (ER) Ca^2+^ release. Extracellular and intracellular Ca2+ was chelated with 1 mM EDTA and 20 PM BAPTA/AM, respectively. Chelation of extracellular rather than ER Ca^2+^ protects against Ivermectin; plate bound viability assay and flow cytometry analysis of

suspension cells are shown. (F) Ivermectin induces generation of ROS in both murine and human TNBC cells. (G) Blockade of H2O2- and Ivermectin-induced ROS by ROS scavengers NAC and GSH. (H) ROS scavengers fail to prevent or delay Ivermectin cytotoxicity.

**Figure 3. Extracellular Dual roles of ATP and purinergic signaling in Ivermectin’s killing.** 4T1.2 cells were treated with Ivermectin for 4h in the presence of different inhibitors at PM concentrations as indicated. (A) The non-specific P2 purinergic receptor inhibitor Suramin (4-400 PM) exacerbates Ivermectin-induced cell death. (B) Inhibition of extracellular ATPases with PSB 069 protects against

Ivermectin cytotoxicity. (C) Blockade of pannexin-1 channels with Panx-1 mimetic blocking peptide

(Anaspec) and Probenecid at PM concentrations as indicated exacerbates killing. (D) Human MDA-MB-

231 cells have higher background membrane permeabilization to YOPRO-1 than the murine 4T1.2 cells and do not respond to further stimulation with 3 mM ATP and Ivermectin. (E) ATP plays a transiently protective role only in the mouse but not human breast cancer cells. 4T1.2 and MDA-MB-231 cells were treated with 32 PM Ivermectin for 4h in the presence of ATP at mM concentrations as indicated. (F) qPCR showing up-regulation of P2X4 and P2X7 receptors on several murine cancer cell lines, including

4T1.2 and DD-Her2/neu (breast), and B16 (melanoma). (G) Up-regulated expression of P2X4 and P2X7 receptors is a characteristic feature of human breast cancer. Expression of P2X and P2Y purinergic receptors, NOX family members, GABA, and Glycine receptors was analyzed using publicly available breast cancer cell lines datasets. Statistically significant (p<0.05) up-regulation and down-regulation in breast cancer versus normal cells is shown in red and blue, respectively. (H) Correlation between sensitivity to Ivermectin and ATP across various human cancer cell lines. Cancer cells were treated for

24h with 3 mM ATP or Ivermectin at 16 and 32 PM doses as indicated. (I) KN-62 (10 PM) blocks both apoptotic and necrotic cell death (IVM 8 or 32 PM, 4h). (J) 10 PM KN-62 provides protection against Ivermectin in a broad spectrum of murine and human cancer cell types. Murine cells were treated with 32

PM Ivermectin for 4h, while human cells were treated with 16 PM Ivermectin for 24h. (K) The P2X7 inhibitors PPADs, oxATP, and A438079 fail to provide protection against Ivermectin. 4T1.2 cells were treated with Ivermectin for 4h after 30 min pre-incubation with P2X7 inhibitors at PM concentrations as indicated. (L) Modulation of ATP+Ivermectin-induced membrane permeabilization by various P2X7- specific inhibitors. Membrane permeabilization in 4T1.2 cells was induced by the combination of 3 mM ATP and 32 PM Ivermectin. P2X7 inhibitors blocked membrane permeabilization at the PM doses indicated.

**Figure 4.** E**xcessive Ca^2+^** /**CaMKII signaling and MPTP contribute to cell death**. (A) Inhibition of

CaMKII blocks initial cytotoxicity in various highly Ivermectin-sensitive mouse cancer cell lines. Murine

breast (DDHer-2), colon (CT26) and prostate (RM1) cancer cells were treated for 4h with 32 PM Ivermectin in the presence of the P2X7/CaMKII dual inhibitor KN-62 or the CaMKII-specific inhibitor KN-93 at PM concentrations as indicated. (B) Inhibition of MPTP with Cyclosporin A protects various highly Ivermectin-sensitive mouse cancer cell lines. Mouse breast (DDHer-2), colon (CT26) and prostate (RMI) cancer cells were treated for 4h with 32 PM Ivermectin in the presence of the MPTP inhibitor Cyclosporin A at PM concentrations as indicated.

**Figure 5. CaMKII-independent P2X7-mediated killing.** (A) P2X7 knockdown inhibits both the early necrotic and the later apoptotic death pathways. (B) Comparison of the protective effects of P2X7, CaMKII, and NADPH oxidase inhibition in short-term (4h, 32 PM IVM) and long-term (24h, 12 PM IVM) exposure of murine versus human TNBC cells. (C) Only inhibition of the P2X7 receptor with KN-

62 rather than inhibition of CaMKII (KN-93) and MPTP (Cyclosporin A) protects against ATP induced

cytotoxicity. 4T1.2 and MDA-MB-231 cells were treated for 24h with 2 and 3mM ATP, respectively, in the presence of various inhibitors (PM concentrations indicated). (D) High concentrations of exogenous extracellular ATP favor the necrotic over apoptotic death pathway induced by Ivermectin. (E) The CaMKII-specific inhibitor KN-93 is the most potent at blocking ATP- and Ivermectin- induced membrane permeabilization to YOPRO-1. 4T1.2 cells were treated with 32 PM Ivermectin for 30 min in the presence of YOPRO-1 and 10 PM KN-62, 30 PM KN-93, and 400 PM Suramin. Asterisk (*) indicates p<0.05 relative to the respective control in each treatment group.

**Figure 6. Ivermectin induces autophagy.** (A) Murine 4T1.2 (breast), but not murine CT26 (colon

adenocarcinoma) or human MB231 (breast) cancer cells manifest constitutive surface exposure of Calreticulin (CRT). (B) Ivermectin does not induce the plasma membrane exposure of CRT (P>0.05) on live MB231 cells, neither does it up-regulate the exposure of CRT on the murine 4T1.2 cells. 4T1.2 and MB231 cells were treated with different doses of Ivermectin for 4h and 24h followed by surface staining with antibody-specific for both mouse and human CRT versus Isotype control and in the presence of

viability/membrane integrity probe. Flow cytometry plots shown are gated on live or dead cells as indicated.

**Figure 7. Ivermectin up-regulates the plasma membrane exposure of Calreticulin (CRT) on live MDA-MB-231 cells.** Tumor cells were treated with different doses of Ivermectin for 24h (6 and 12 μM IVM) and 2h (8, 16, and 32 μM IVM), as indicated. Cells were surface-stained with polyclonal rabbit anti-CRT antibody (Abcam, ab2907) versus rabbit IgG polyclonal isotype control (Abcam, 171870), followed by staining with AF488-labelled donkey anti-rabbit IgG secondary antibody (Biolegend, 406416). Viability/membrane integrity probe was also included to discriminate between live and dead cells. (A) A representative overlay histogram plot on the left demonstrates up-regulation of CRT on gated live cells treated with 12 μM IVM for 24h. (B) Bar plots show a summary of the ratios of mean fluorescence intensity (MFI) values versus isotype control calculated after gating on live/membrane-intact cells. Up-regulation of CRT on gated live cells was also observed in the murine 4T1.2 and CT26 cancer lines (data not shown).
